# Precise Tuning of the Nanostructured Surface leading to the Luminescence Enhancement in SrAl_2_O_4_ Based Core/Shell Structure

**DOI:** 10.1038/s41598-017-00541-w

**Published:** 2017-03-28

**Authors:** Rocío Estefanía Rojas-Hernandez, Fernando Rubio-Marcos, Aida Serrano, Adolfo Del Campo, José Francisco Fernandez

**Affiliations:** 1 Electroceramic Department, Instituto de Cerámica y Vidrio, CSIC, Kelsen 5, 28049 Madrid, Spain; 2 SpLine, Spanish CRG Beamline at the ESRF, F -38043, Grenoble, Cedex 09 France; 3 Instituto de Ciencia de Materiales de Madrid, CSIC, Cantoblanco, 28049, Madrid Spain

## Abstract

Intensive research has been focused on the synthesis of long-lasting SrAl_2_O_4_:EuDy in luminescent materials field. Traditionally, SrAl_2_O_4_:EuDy is synthesized in bulk form by solid state. However, their development remains restrained due to this technique is not compatible with large-scale production, sustainability and nanometer-scale requirements. Despite nano-range particles have been obtained by chemical routes, photoluminescence response decreases and application became unpractical. It remains a challenge to synthesize nonrare-earth (RE) phosphors with high photoluminescence. One major challenge for the luminescent materials community is to devise methods to deliver innovative, sustainable and cost effective solutions for the reduction of RE because of the lack of RE availability. Here, we suggest a solution based on molten salts, obtaining nanosheets or micro/nanostructured SrAl_2_O_4_:Eu, Dy particles with core-shell structure, employing only 50% of standard amounts of RE. Core-size and shell thickness and crystallinity can be tuned by post-thermal treatment, through which can be modulated the Eu^+2^ fraction. We find that our methodology leads the functional features of the analogous micron counterpart. These results can be considered a great achievement to scale-up the process. Besides, the harmful collateral effect of nanotechnology must be addressed by using new safe by design core-shell nanostructures.

## Introduction

The research growth and breakthrough in luminescence materials, also called phosphors, has had a rapid progress due to their widespread applications in the past decades. In spite of lately organic luminescent materials have acquired notable interest; luminescent materials are mostly inorganic materials due their interesting physics properties and stability. Depending on the persistent of the luminescence, phosphors can be classified in fluorescent if light emission remains 10^−9^–10^−7^ s after the excitation and phosphorescent when the life time is higher^1^. Long-lasting afterglow SrAl_2_O_4_:Eu, Dy powders remain the phosphorescent material more extensively studied and used due to their brightness and persistence^2^.

Generally speaking, SrAl_2_O_4_:Eu, Dy powders consist mostly of micrometer-sized particles; being the average particle size typically within the 20–100 μm range and the crystallite size above 15 nm^3, 4^. Large scale production requires temperatures >1500 °C for long-time, >10 h, which results in a large particles growth. The actual size of long lasting phosphors is limiting for current applications that demand sub-micron particles as digital printing. Top-down processes result deleterious for the optical properties in the strontium aluminate based phosphors, meanwhile bottom-up nanomaterials fails to obtain the great photoluminescence response requires for practical applications^5^. At this point it is necessary to point out the lack of comparative measurements in most of the studies because of the extent use of arbitrary units to show up the luminescence response.

Luminescent nanoparticles are under extensively study due to quantum confinement effect which leads to novel optoelectronic properties. However, in terms of quantum efficiency the main disadvantage of nanosized phosphors is the increasing of defects number due to the larger surface/volume ratio. In addition, the crystal size has also a great influence on the photoluminescence properties. For example, the absorption of excitation light is decreased by the strong light scattering of nanocrystals and therefore the reduction of the intensity emission.

Mostly, inorganic matrices are doped with activator cations; basically the doping cations are rare earth elements and in lesser extent transition metals^6^. By now, the 80% of known persistent phosphors are based on rare-earths^7, 8^. However, because of the lack of rare earth availability on the market dominated by China, and the environmental hazards of their mining and processing pushed the community to explore novel non-rare earth doped phosphors^9, 10^. Currently, it remains a challenge to synthesize non-rare-earth phosphors with high photoluminescence through a convenient chemical route. Nowadays, the persistent luminescent research should deliver innovative, sustainable and cost effective materials solutions for the reduction of rare earth elements used in luminescent materials due their scarcity^11^.

Therefore, different strategies are still a challenge in this field. Among others, some routes should be mentioned: to synthesize defect-free nanopowders with high crystallinity; as to decrease the rare earth content in already existing materials; or to improve photoluminescence response by encapsulation using capping agents. In general the expected photoluminescence jump has not been achieved yet^12^. Therefore, new approaches are being required to overcome these synthesis limitations. Over time, various synthesis methods emerged, for instance, microemulsion and molten salt synthesis. On one hand, by usual microemulsion process^13^, it is necessary to carry out an annealing process in reducing atmosphere and by a double- thermal treatment at 1000 and 1100 °C, where the obtained particles grow significantly, ca. 3–50 μm. On the other hand, molten salt process possesses relevant advantages over the previous methods for the synthesis of strontium aluminates, the main one being that synthesis in flux is potentially interesting to obtain high crystallinity and therefore large luminescent response.

The starting alumina on the synthesis by molten salt has an influence in the kinetic of the reaction. The reactivity of the starting alumina is modulated by their nature, size and morphology. Therefore, SrAl_2_O_4_ formation, their final size and morphology depend on the reactivity of alumina precursor. Employing as precursor reactive alumina (Almatis, Specific Suface Area, BET: 13 m^2^/g, average particle size, d_50_ ≈ 0.1 μm) high crystallinity submicron SrAl_2_O_4_:Eu, Dy particles have been obtained heated at 1000 °C for 2 h in 90N_2_-10H_2_ atmosphere, employing salt/SrAl_2_O_4_ molar ratio of 3:1^14^. These particles have a better photoluminescence response in comparison with a commercial powder based on SrAl_2_O_4_:Eu, Dy mechanically conditioned to achieve a particle size down to the one available in the market. The powder synthesized by molten salt method has a higher photoluminescence response and a suitable relationship between functional properties and the particle size^14, 15^. However, their response could not achieve the features of standards requires for long lasting phosphors. For the purpose of obtaining submicron particles, the synthesized nanomaterials have usually spherical morphology. However, other morphologies such as flat nanosheets, elongated nanowires, fibers or rods may be interesting to enhance the luminescence response. Molten salt synthesis can be considered as an attractive strategy to generate these kinds of morphologies, when the technique develops a template assisted approach. Micro/nanostructured materials in the form of microscale particles assembled from nanoscale elements can keep away from shortcomings of nanomaterials and retain the inherit advantages. Self-supported micro/nanostructures design onto microscale particles as building blocks would be a good direction to improve the performance of phosphorescent materials^6^.

Herein, we present a practically scalable approach for surface nanocrystallization of SrAl_2_O_4_:Eu, Dy in an alumina’s template. With a simple synthesis by molten salt of SrAl_2_O_4_:Eu, Dy, the entire surface of alumina’s template was readily converted into core-shell structure, which enables a drastic reduction of rare earths to obtain a superior photoluminescence response than the commercial materials. Through this novel design, we have found a linked relationship between the % of photoluminescence intensity and the Eu fraction. The core-size, the thickness and crystallinity of the shell can be tuned and we have further shown the flexibility of our approach by post-synthesis treatment controlled, through which can also be easily modulated the Eu^+2^ fraction. Our study provides an effective way to shorten the gap between conceptual and experimentally available SrAl_2_O_4_:Eu, Dy materials, and therefore drives the development of new classes of technologically relevant phosphorescent materials.

## Results and Discussion

The SrAl_2_O_4_:Eu, Dy powders have pseudo-spherical morphology and particle size ≤0.5 μm when a sub-micron Al_2_O_3_ (0.1 μm Al_2_O_3_) is employed^14, 16^. This can be attributed to a higher reactivity in the system and the dominance of dissolution-precipitation mechanism. However, the use of larger alumina, 6 μm Al_2_O_3_, here employed, modifies the reaction pathway leading to a different reaction evolution.

Optimizing the Al_2_O_3_/SrO ratio of the SrAl_2_O_4_:Eu, Dy powders synthesized by the molten salt method with 6 μm Al_2_O_3_ as a precursor, the needed secondary phases and the concentration of dopant can be reduced keeping the photoluminescent response of the synthesized powder (see Supporting Information, Fig. S1).

**Figure 1:**
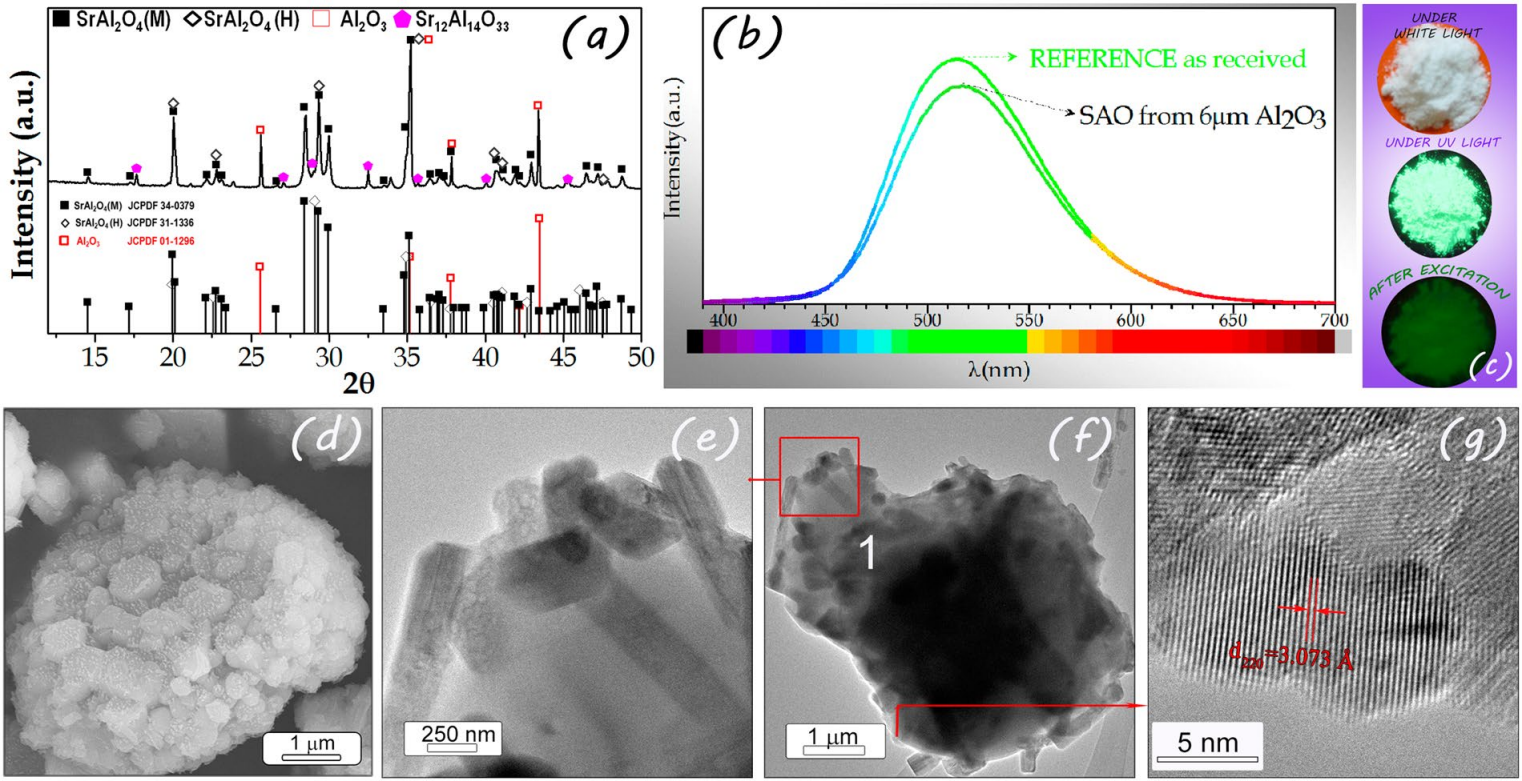
Structural, Microstructural, Morphological Characterization of the powder synthesized and their photoluminescent response. (**a**) XRD pattern of synthesized SrAl_2_O_4_:Eu, Dy (SAO) phosphor heated at 1000 °C for 2 h in 90N_2_-10H_2_ atmosphere, employing a salt/SrAl_2_O_4_ molar ratio of 3:1 as precursor an alumina with an average particle size, d_50_ ≈ 6 μm. The symbols highlight Al_2_O_3_ (red-open squares), SrAl_2_O_4_ (Hexagonal) (black-open diamonds), and SrAl_2_O_4_ (Monoclinic) (black squares). The standard diffraction pattern (JCPDS 34-0379) of SrAl_2_O_4_ monoclinic polymorph, (JCPDS 31-1336) of SrAl_2_O_4_ hexagonal polymorph and (JCPDS 01-1296) of Al_2_O_3_ are also plotted at the bottom of the figure. (**b**) Emission spectra upon excitation at 380 nm of synthesized SrAl_2_O_4_:Eu, Dy detailed in Fig. 1(a) and compared with the commercial powder (from Jinan Chenghao Technology Co., Ltd) as received. (**c**) Photographs of the powders under white light excitation (upper-image), under UV light excitation (middle-image) and in dark after being stimulated with light (bottom-image). (**d**) FE-SEM and (**e–g**) TEM and HRTEM micrographs of the synthesized powders.

Figure 1(a) shows the XRD pattern of the powder synthesized by the molten salt method heated at 1000 °C for 2 h in 90N_2_-10H_2_ atmosphere, employing a salt/SrAl_2_O_4_ molar ratio of 3:1 and Al_2_O_3_/SrO ratio of 2, an alumina with an average particle size, d_50_ ≈ 6 μm. The XRD shows characteristic peaks of the SrAl_2_O_4_ monoclinic polymorph (space group P2_1_)^17^, whose pattern is characterized by three peaks centered in the 2θ range from 28° to 30° and matched with the SrAl_2_O_4_ standard values given in JCPDS (No. 34-0379) and their coexistence with SrAl_2_O_4_ hexagonal polymorph (space group P6_3_22), which exhibits a main peak at 2θ = 29.06°. XRD pattern reveals the presence of other phases, which could be identified as Al_2_O_3_ (JCPDF file 73-1512) and Sr_12_Al_14_O_33_ (JCPDF file 40-0025). Taking into account the reaction mechanism established previously^16^ and the results in Supporting Information, Figs S1–S2b, the most probable origin of rest of raw materials can be related to unreacted Al_2_O_3_ core.

Figure 1(b) shows the photoluminescence emission spectrum (λ_exc_ = 380 nm) of SrAl_2_O_4_:Eu, Dy powder compared to a commercial powder (from Jinan Chenghao Technology Co., Ltd) as received, which has an average particle size, d_50_ ~ 20 μm. The emission band centered at 515 nm is assigned to the transition of 4f^6^5d^1^ → 4f^7^ (^8^S_7/2_) of Eu^2+^ ions^18^. The emission intensity of the powder synthesized by molten salt is close to that of the commercial powder but it is still lower; However it should be taken in account that the commercial powder has particles larger than 20 μm while the powder synthesized by molten salt has particle size lower than 10 μm as is shown in SEM characterization. The chemical composition of the commercial powder was determined by X-ray Fluorescence (XRF) (See Supporting Information Table S1). From these values, molar concentration of Europium and Disprosium was calculated; being c.a. 0.02 to Eu and c.a. 0.01 to Dy. For this reason, we have employed this reference product to develop a accurately comparative analysis with the powder synthesized in our laboratory, that has the following composition *Sr*_*1*−*x*−*y*_ *Eu*_*x*_ *Dy*_*y*_ *Al*_*2*_ *O*_*4*_ *with x* = *0*.*02 and y* = *0*.*01*. Figure 1(c) shows three photographs of the as resulted powders after the synthesis process under different excitation source. As one can see in the upper photo an interesting white powder is obtained under artificial indoor illumination, in comparison with commercial yellowish phosphors. The white colour of the powder could be attributed to the roughness of the nanostructured surface that could contribute to light scattering. The image in the middle shows a photograph of the resultant powders emitting in the green phosphorescent region under UV illumination and the bottom image in darkness after cutting of the excitation.

Figures 1(d) illustrates the micrograph by field emission scanning electron microscopy (FE-SEM) of the synthesized powders, finding that the particles retain the shape of the original Al_2_O_3_. More specifically, the growth of SrAl_2_O_4_:Eu, Dy sub-micron particles on the surface of hexagonal platelets of 6 μm Al_2_O_3_ is promoted. These nanostructured sheets have a particle size c.a. 10 μm with a thickness ≤2 μm, which it is an interesting dimensional size for many applications.

High-resolution transmission electron microscopy (HRTEM) was performed to obtain a detailed phase and morphological characterization. TEM and HRTEM analyses of the powder are illustrated in Fig. 1(e–g). The low magnification TEM image (Fig. 1(f)) shows the presence of hexagonal platelets with a mean particle size of ca. 8 μm. The magnified micrograph is shown in Fig. 1(e). There are rod-shape in section particles that come from the synthesized nanostructured SrAl_2_O_4_:Eu, Dy. Theses surface particles consist of well crystallized nanoregions (Fig. 1(g)). The interplanar spacing was calculated as 3.073 Å from the inverse FFT pattern, matching to the (220) (d_222 Theoretical_ = 3.048 Å) reflection of the SrAl_2_O_4_ pattern (JCPDS no. 34-0379). The EDS shows that the chemical components of the samples are the elements Sr, Al, O, Eu and Dy. This analysis confirmed that the atomic % of the elements has a close agreement between the theoretical and calculated value, as shown in Table 1.

**Table 1:** Atomic % of the elements Sr, Al, O, Eu and Dy in the synthesized SrAl_2_O_4_:Eu, Dy phosphor heated at 1000 °C for 2 hour in 90N_2_-10H_2_ atmosphere, employing a salt/SrAl_2_O_4_ molar ratio of 3:1 as precursor alumina platelets with an average particle size, d_50_ ≈ 6 μm) and using SrAl_2_O_4_/SrO molar ratio of 2.

Atomic (%)	Sr	Eu	Dy	Al	O	Total
Teoric (SrAl_2_O_4_:Eu, Dy)	13,9	0,3	0,1	28,6	57,1	100
Point 1	16,73	0,00	0,00	30,59	51,71	100
Uncertainty	0,31	100,00	100,00	0,14	0,19	100

Bearing in mind the results previously obtained in the design of nanostructured SrAl_2_O_4_:Eu, Dy system synthesized by molten salt, it is important to remark that the reduction of temperature, the duration of the thermal treatment and the needed precursors are achieved. The optimization of the Al_2_O_3_/SrO ratio to 2 involves the reduction of SrO compared with stoichiometric ratio. Europium and dysprosium substitute strontium, so their content is notably reduced; only 50% of the standard amounts, as a consequence a relevant reduction of rare earths occurs; following this composition *Sr*_*1*−*x*−*y*_ *Eu*_*x*_ *Dy*_*y*_ *Al*_*2*_ *O*_*4*_ *with x* = *0*.*01 and y* = *0*.*005*. As a result, the viability, suitability and scalability of the synthesis strategy are demonstrated.

**Figure 2:**
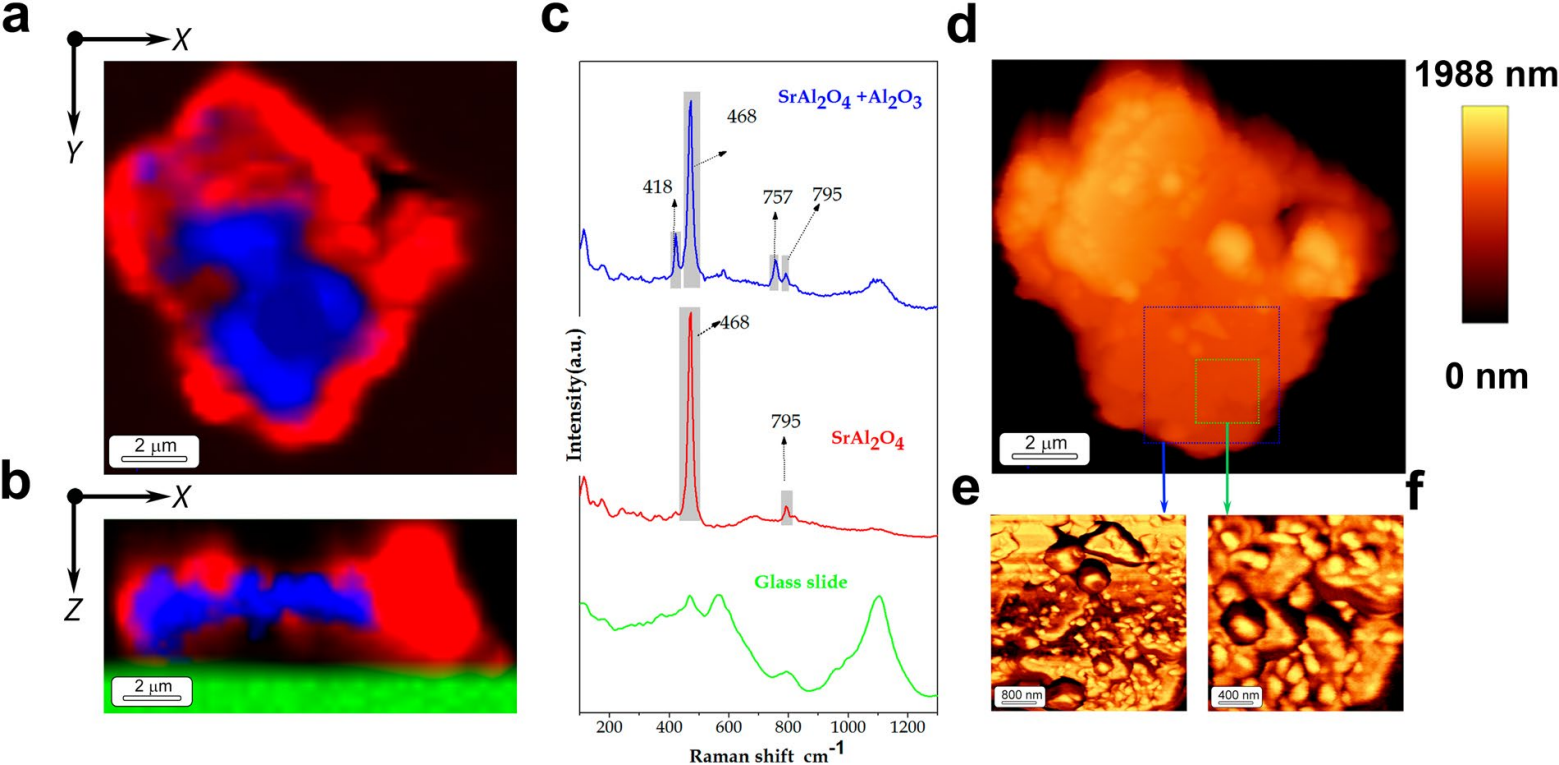
Corroborating core-shell structure by Coupling Confocal Raman and AFM technique. (**a**) XY and (**b**) XZ Raman image of the platelet based on SrAl_2_O_4_:Eu, Dy phosphor heated at 1000 °C for 2 h in 90N_2_-10H_2_ atmosphere, employing a salt/SrAl_2_O_4_ molar ratio of 3:1, as precursor an alumina platelets with an average particle size, d_50_ ≈ 6 μm and Al_2_O_3_/SrO molar ratio of 2. (**c**) Average Raman spectra correspond to each phase; the colour-coded Raman spectrum corresponds with the colour-coded areas in Raman images. (**d**) The AFM topography of the same scanning area in Raman study. (**e**,**f**) AFM phase imaging micrographs of the areas selected on Fig. 2(d).

From XRD, we can state the reminiscence of unreacted Al_2_O_3_ in presence of SrAl_2_O_4_ phase. However, from SEM and HR-TEM discrete alumina has not been observed. By molten salt route, there are usually two main mechanisms: (*i*) dissolution-precipitation mechanism; and (*ii*) dissolution−diffusion transport mechanism (“template mechanism”). The relative dissolution rate determines the dominant formation mechanism. This template mechanism could explain the reaction pathway that leads a core-shell structure, which will be formed by shell-like SrAl_2_O_4_ formation and a core of Al_2_O_3_, as first approximation. To study this superficial growth when 6 μm platelets are employed and to verify that the outer layer is based on SrAl_2_O_4_ and the inner part based on Al_2_O_3_, discrete platelets Coupling Confocal Raman and AFM technique have been employed. The coupling of these techniques allows establishing core-shell structures which cannot be determined by other techniques. Platelets dispersion was carried out in absolute ethanol by ultrasonic agitation; subsequently the platelets were settled into a glass slide. Raman study was carried out by means of the XY and XZ depth Raman images coupled with AFM. Considering that the platelet core should be evaluated, the focus was located 0.5 μm below the particle surface to acquire the XY Raman image, Fig. 2(a). These Raman images show a colour-coded indicating the region of the sample where the Raman spectrum corresponds to the one presented in the Fig. 2(c). Therefore, the XY (Fig. 2(a)) an XZ (Fig. 2(b)) Raman image provides information about the spatial distribution of the different phases, distinguishing two areas. Both spectra show the Raman peak around 467 cm^−1^ attributed to the bending of O-Al-O bonds of SrAl_2_O_4_ phase^19, 20^. In addition, there is a Raman mode less intense at 795 cm^−1^ assigned to SrAl_2_O_4_ phase^16^. The difference between the spectrum of these areas is the presence or the absence of the intense Raman mode located at 418 cm^−1^ and the low intense Raman mode located at 757 cm^−1^, which are attributed to A_1g_ and E_g_ α-Al_2_O_3_ (corundum) phonon modes, respectively^21^. These findings suggest that the strontium aluminate particles possess an unreacted Al_2_O_3_ core and reveal the formation of a core-shell structure formed by a rich Al_2_O_3_ core and a SrAl_2_O_4_ shell, as shown in Fig. 2(a). To confirm the rich Al_2_O_3_ core, a depth Raman image has been carried out (Fig. 2(b)). As illustrated Fig. 2(b) the discrete platelet has a thickness ≤2 μm, the SrAl_2_O_4_ phase prevails in the outer part and the Al_2_O_3_ phase predominates in the inner part. The green area represents the slide glass, whose spectrum is exhibited in Fig. 2(c). Further measures based on Raman study by means Raman depth profiles indicate the formation of a core-shell structure (Fig. S2, Supporting Information). In addition, these measures clearly disclose shell–like formation based on SrAl_2_O_4_ and confirm the thickness of the SrAl_2_O_4_ shell that is ≈500 nm.

Additionally, an AFM topography study was carried out. The AFM image of the same scanning area is shown in Fig. 2(d). The surface topography reveals the nanocrystalline nature of the surface layer. Besides, the growth of surface nanostructures based on SrAl_2_O_4_:Eu, Dy was observed. Figure 2(e,f) present AFM phase imaging of areas marked on Fig. 2(d), visualizing a higher contrast of nanostructures. This nanostructure formation process is driven by the dissolution−diffusion transport mechanism. The nanoparticles reveal sizes ranging from 40 to 800 nm, with most particles at 250 nm.

**Figure 3:**
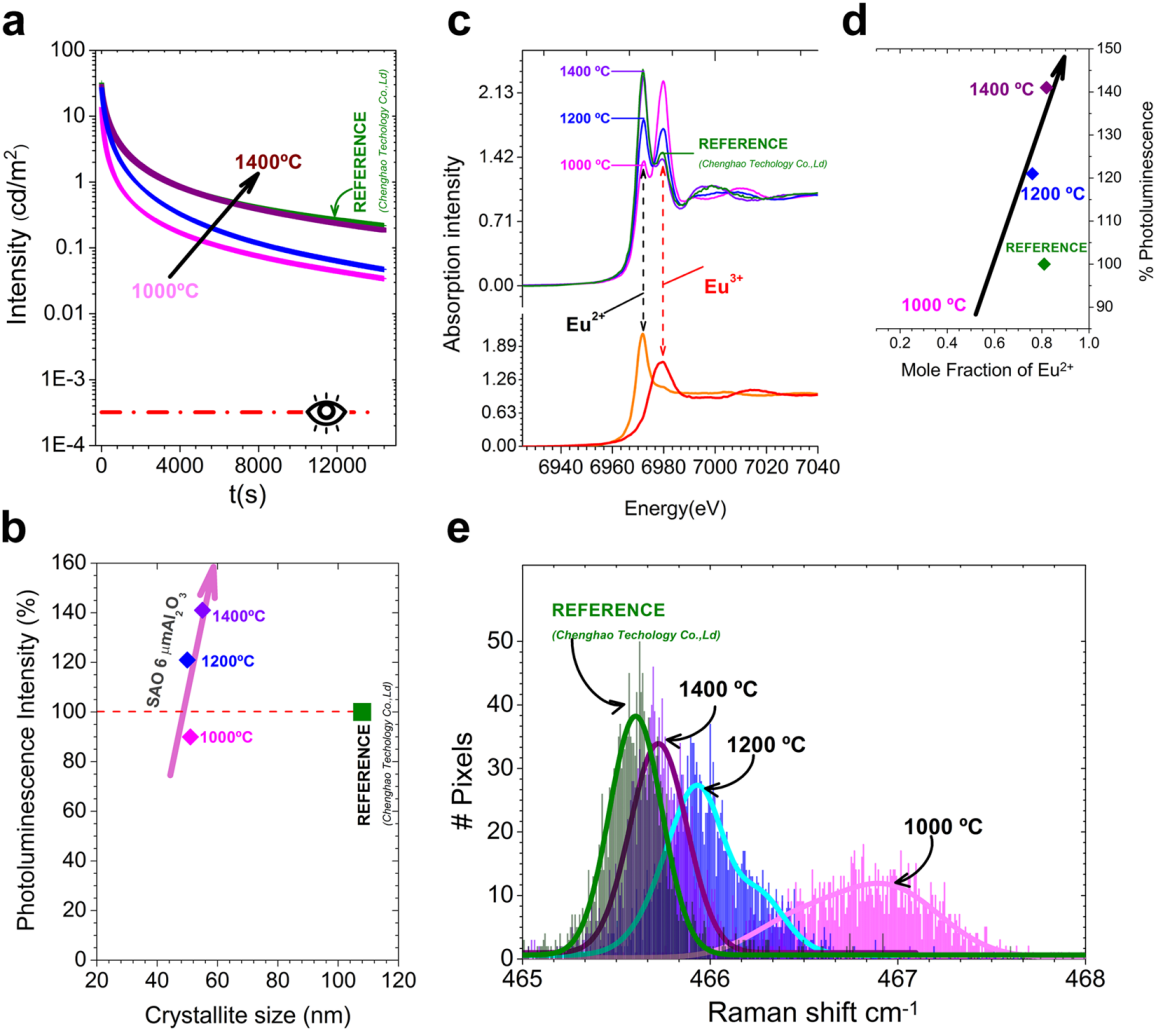
Functional properties can be tuned by post-thermal treatment. (**a**) Afterglow decay curve of SrAl_2_O_4_:Eu, Dy powder synthesized at 1000 (magenta decay curve), 1200 (blue decay curve), and 1400 (purple decay curve) °C for 2 h in 90N_2_-10H_2_ atmosphere, employing a salt/SrAl_2_O_4_ molar ratio of 3:1, as precursor alumina platelets with an average particle size, d_50_ ≈ 6 μm) and using Al_2_O_3_/SrO molar ratio of 2 compared to the commercial powder (green decay curve) after the light source was cut off, irradiated for 10 min by solar simulator. (**b**) % of photoluminescence intensity tendencies taking as reference the commercial powder as a function of the crystallite size for the initial reference material and the materials synthesized here by means of molten salts referred to as SAO 6 μm Al_2_O_3_ (Al/Sr: 2) synthesized at 1000, 1200 and 1400 °C. (**c**) Top panel: XANES spectra of the powder synthesized at 1000 (magenta spectrum), 1200 (blue spectrum), 1400 °C (purple spectrum) and initial reference material: microparticle SrAl_2_O_4_:Eu, Dy from Jinan Chenghao Technology Co., Ltd (green spectrum). Bottom panel: XANES reference spectra of Eu^2+^ (EuI) and Eu^3+^ (Eu_2_O_3_). (**d**) % of photoluminescence intensity of SrAl_2_O_4_:Eu, Dy powder synthesized at 1000 (magenta diamond), 1200 (blue diamond), 1400 °C (purple diamond) and initial reference material (green diamond). As a function of the Eu^2+^ fraction. (**e**) Statistic distribution of the Raman shift for O-Al-O Raman mode for of SrAl_2_O_4_:Eu, Dy powder synthesized at 1000 (magenta distribution), 1200 (blue distribution), 1400 °C (purple distribution) and initial reference material (green distribution).

In academia there is not a standardized way of measuring and defining persistent phosphors properties. The German norm DIN 67510-1, the most used in commercial field, employs as irradiation source a Xe Arc Lamp (1000 lux). However, unfiltered Xe-arc take in account a great contribution of short ultraviolet radiation that is completely different in comparison to solar or artificial indoor illumination. Nonetheless, the majority of decay curves are taken when the sample is irradiated with monochromatic light at 350, 365 and 375 nm for 5 and 10 min^22–29^ and by a solar simulator in a lesser extent^30^. Usually, arbitrary units are employed to measure the photoluminescence intensity and the afterglow decay, so it is difficult to compare experimental measures obtained by different research groups. There is a standard to measure the emission of light in cd/m^2^, but it is arguable due to eye sensitivity becomes more blue-sensitive at lower light levels and the photometric standard does not span the scotopic-mesopic-photopic spectrum^31, 32^. Figure 3(a) shows the afterglow decay curve of synthesized SrAl_2_O_4_:Eu, Dy powder compared to the commercial powder after the light source was cut off, irradiated for 10 min by a solar simulator. Other sources have been also employed (see Supporting Information, Fig. S3). The phosphorescent decay time follows an exponential law^33^. Although the synthesized powder shows a lower emission and afterglow initial intensity than the commercial counterpart, the reduction is less than the drop reported by Tang *et al*., which decreases by 1 order of magnitude. In addition, the afterglow intensity at 4 h is above the visibility threshold or the limit light perception of dark-adapted human eye, 0.32 mCd/m^2^ (100 times the light perception of the scotopic vision)^31^. A wide range of applications can be sought because of SrAl_2_O_4_:Eu, Dy is a promising candidate for radiation detection. According to the scintillation properties of SrAl_2_O_4_:Eu, Dy powder, we evaluated also the X-ray response (see Supporting Information, Fig. S4).

One of our aim is synthesizing functional materials based on SrAl_2_O_4_:Eu, Dy at low temperature, and effectively employing as precursor alumina platelets with an average particle size, d_50_ ≈ 6 μm, using Al_2_O_3_/SrO molar ratio of 2. Powders with better properties than the powders obtained until now are obtained. In addition, high temperatures, 1200 and 1400 °C, are evaluated to analyze their role in the final properties.

The afterglow decay curves of SrAl_2_O_4_:Eu, Dy powder synthesized at 1200 and 1400 °C are shown in Fig. 3(a). The decay process of luminescence undergoes an initial fast decay followed by a slow decaying process. However, the initial fast decay decreases with the thermal treatment increment.

Previously, it has been established that the photoluminescence intensity is related with the extrinsic (*i*.*e*. *morphology*, *particle size*) and intrinsic features of the particles, but its reduction has a great influence mainly on the intrinsic characteristic of the particles (*i*.*e*. *crystallinity*)^15^. Figure 3(b) illustrates the % of photoluminescence intensity taking as reference the commercial powder as a function of the crystallite size for the initial reference material the materials synthesized by means of molten salts using as precursor alumina with an average particle size, d_50_ ≈ 6 μm referred to as SAO 6 μm Al_2_O_3_ (Al/Sr: 2) synthesized at 1000, 1200 and 1400 °C. Figure 3(b) tries to elucidate different tendencies related to the % of photoluminescence as a function of the crystallize size.

Generally speaking, the photoluminescence intensity decreases with crystallite size reduction^15, 16^. However, the above results clearly show that the material synthesized by molten salts route SAO 6 μm Al_2_O_3_ (Al/Sr: 2)) has a large photoluminescence in spite of their lower crystallite size in comparison with the reference. Obviously, once again the photoluminescence increases with the crystallite size if we compare the powders synthesized by molten salt route a higher temperatures.

It is important to remark that the emission peak attributed to the 4f^6^5d^1^ → 4f^7^ (^8^S_7/2_) of Eu^2+^ ions^18^ transition became more intense with increasing the temperature(from 1000 to 1400 °C); the % of photoluminescence is higher as shown in Fig. 3(b). According with these data by the core-shell approach only 1/3 of the rare earth content is required to obtain the same photoluminescence than the commercial phosphor. To quantify the relative amounts of the two oxidation states for Eu element, X-ray absorption near-edge structure spectroscopy (XANES) measurements at the Eu L_3_ edge were performed. The reference spectra of Eu^2+^ (EuI) and Eu^3+^ (Eu_2_O_3_) are shown in Fig. 3(c) (bottom panel). A significant difference in the energy can serve as a signature of the two oxidation states. The spectra of the powder synthesized at 1000 (magenta spectrum), 1200 (blue spectrum), 1400 °C (purple spectrum) for 2 h in 90N_2_-10H_2_ atmosphere, employing a salt/SrAl_2_O_4_ molar ratio of 3:1 and alumina platelets (average particle size, d_50_ ≈ 6 μm) and using Al_2_O_3_/SrO molar ratio of 2 and the commercial powder (green spectrum) are shown in Fig. 3(c) (top panel). These spectra consist of two peaks coinciding with the reference spectra of Eu^2+^ and Eu^3+^, definitively confirming the coexistence of both oxidation states. High-temperature annealing results in a notable reduction in the Eu^3+^ and an increase in Eu^2+^ peak intensity. It should be emphasized that the % of photoluminescence intensity increases accordingly with the Eu^2+^ fraction, as seen in Fig. 3(d) (see Supporting Information, Fig. S5 and Table S2). The location of the nanostructured strontium aluminate at the shell region favors the diffusion kinetic during the thermal treatment under reduction atmosphere. Therefore, high temperatures increase the photoluminescence response compared with the reference powder due to the Eu^2+^ content in Fig. 3(c,d), mainly, and the higher crystallinity as second factor (see Supporting Information, Fig. S6). The coexistence of Eu^2+^ and Eu^3+^ and the ability to control their relative fraction allow for the design and preparation of new light emitting materials. Even though the Eu^2+^ fraction of the commercial powder is similar than the powder synthesized at 1400 °C, the photoluminescence response is lower due to the Eu^2+^ is localized inside the large particles and not contributes in the emission. Nanosheets or micro/nano-structured SrAl_2_O_4_:Eu, Dy in the form of microscale particles assembled from nanoscale crystals allow a better europium distribution.

Stimulated by the above issue and considering that the Raman mode around 466 cm^−1^ is attributed to the bending of O-Al-O bonds of SrAl_2_O_4_ phase. The Raman shift image allows us evaluating the homogeneity on the sample because each pixel on the Raman image comprises a full Raman spectrum (see Supporting Information, Fig. S6). The statistical analysis of Raman shift of the O-Al-O mode Raman shift of all spectra in the image is shown in Fig. 3(e). This statistical distribution of the Raman shift regarding the main Raman mode shows a broad distribution related to the coexistence of hexagonal and monoclinic phase and the lower crystallinity in the sample synthesized at 1000 °C. The signal corresponding to the powder synthesized at 1200 °C splits into two distributions band that reveal the presence of two phases that is correlated with XRD results. There is a large red shift of the Raman modes related to O-Al-O bonds and the distribution can be considered to be approximately Gaussian in the sample synthesized at 1400 °C. This red-shift of the Raman modes indicates a decreasing of the bond constant force that correlated with the larger ionic size of the Eu^2+^, 1.25 Ǻ, than the Eu^3+^, 1.07 Ǻ, that result in a relaxation of the crystal lattice. The mean value of the distribution is 465.73 cm^−1^. This distribution also reveals unequivocally the homogeneity in the sample with higher photoluminescent response that possesses higher Eu^2+^ content and lower Raman shift. Once again, despite the commercial powder shows homogeneity due to these material is formed for large and crystalline particles synthesized at high temperatures (>1350 °C), their photoluminescence response is lower than the powders synthesized by molten salt.

To sum up, if a top-down strategy is developed to decrease the particle size, the final material is formed by pieces from a larger structure. It is possible to obtain small particles sizes with well-distribution, but the as-obtained phosphor showed a much lower photoluminescence intensity and shorter persistent time compared with the corresponding reference material. Therefore, the solution could be the development of traditional approaches for the synthesis of nano-sized SrAl_2_O_4_:Eu^2+^, Dy^3+^ material called as bottom-up methods such as combustion synthesis, sol-gel, hydrothermal, microemulsion synthesis. Therefore, once again the photoluminescence response decreases. Moreover, other disadvantages appear including multistep procedures that increase the cost of the material, complicated synthesis routes and the requisite of post-thermal treatments. For this reason, a nano-architecture strategy, here developed, could be a potentially approach to synthesize particles with better performance. Following an overview of principles which guide nanocrystal formation, emerging design criteria are outlined toward shape-controlled nanocrystals prepared by molten salt synthesis, a synthetic strategy which spatially and temporally confines crystal growth. Employing a larger and less reactive Al_2_O_3_, well-defined morphology was developed by a template assisted technique, where nanostructures are self-supporting in alumina core.

To conclude, the synthesis of SrAl_2_O_4_:Eu^2+^, Dy^3+^ core-shell structures by molten salts demonstrates a remarkable route to obtain self-supported micro/nanostructures directly. We have described the development of novel micro-sized core–shell particles that have an excellent photoluminescence response. These novel nanoarchitectures are SrAl_2_O_4_:Eu^2+^, Dy^3+^ nanocrystals self-supporting in an alumina’s core and displays superior photoluminescence response than the commercial materials. There is a clear correlation between the % of photoluminescence intensity increment and the Eu^+2^ fraction. A drastic reduction of rare earth is demonstrated employing 50% of the rare earth content. It is worth pointing out that the economical and innovative preparation strategy presented here, can be broadly applied to other compounds exhibiting persistence luminescence to drive the development of new classes of technologically relevant phosphorescent materials. We believe that core-shell structures provide numerous opportunities to facilitate optical detection of secured patterns. The possibility to generate inks and create micrometer sized quick response codes through assemblies of core-shell is a promise strategy for security printing field^34^.

## Methods

### Materials

Al_2_O_3_ (Vicar S.A., 99.5%, average particle size, d_50_ ≈ 6 μm), SrCO_3_ (Merck, 99.9%, d_50_ ≈ 1.1 μm), Eu_2_O_3_ (Metall Rare Earth Limited, 99.5%, d_50_ ≈ 3.8 μm) and Dy_2_O_3_ (Metall Rare Earth Limited, 99.5%, d_50_ ≈ 3.1 μm), were used as precursors.

### Synthesis of sub-micron particles

SrAl_2_O_4_:Eu^2+^, Dy^3+^ particles were synthesized by molten salt synthesis. In all the experiments, the raw materials were first dried at 120 °C for 1 h due to their hygroscopic nature. Sr_1−x−y_Eu_x_Dy_y_Al_2_O_4_ compositions with *x* = 0.02 to 0.01 and *y* = 0.01 to 0.05 were prepared. The molten salt was composed of a mixture of NaCl (99.5% purity) and KCl (99.5% purity) of using a 0.5:0.5 molar ratio (eutectic mixture hereafter abbreviated as *(NaCl-KCl)*_*e*_. It was dry homogenized by grinding in a 60 cm^3^ nylon container for 20 minutes by using a turbula-type mixer at 50 rpm with ZrO_2_ balls with a diameter of 0.5 mm. The above materials were mixed in the same dry conditions using the turbula-type mixer. The salt/SrAl_2_O_4_ molar ratio was kept at 3:1. The homogenized mixture was placed in an alumina crucible with a platinum foil to avoid reaction with the crucible. The powders were heated to a given temperature (1000, 1200 and 1400 °C) in 90N_2_-10H_2_ atmosphere and held for 2 h in order to reduce the Europium and obtain a phosphorescent material. A white powder was finally obtained.

### Structural and Microstructural Characterization

The crystalline phases were characterized by X-ray diffraction (XRD, D8, Bruker) using a Lynx Eye detector and a Cu Kα_1,2_ radiation. Structural characterization has carried out by confocal Raman microscopy coupled with an atomic force microscopy (AFM) instrument (Witec ALPHA 300RA with a Nd:YAG laser excitation at 532 nm at room temperature (RT) and a 100X objective lens (NA = 0.95). The incident laser power was 19.6 mW. The optical diffraction resolution of the confocal microscope was limited to about 200 nm laterally. Raman spectral resolution of the system is down to 0.02 cm^−1^, under the best measurements conditions. The piezoelectric scanning table allows three-dimensional displacements in steps of 3 nm, giving a very high spatial resolution for both the AFM and confocal Raman microscopy. The Raman images consist of ~2400 simple spectra of 1 s of integration time each, so that the measurement time of an image is ca. 40 min. The AFM measurements have been carried out in the noncontact mode, with silicon tip of 285 kHz resonant frequency and 42 N/m force constant. The microscope base is also equipped with an active vibration isolation system, active in the range 0.7–1000 Hz. The microscopy sample was mounted in a piezo-driven scan platform having a 4 nm lateral accuracy. Samples were deposited on a microscopy glass slide. Collected spectra were processed and analyzed by using Witec Control Plus software 2.08. The morphology of the nanostructured powders was evaluated using secondary electron images of field emission scanning electron microscopy (FE-SEM, Hitachi S-4700). The particle size and morphology at the nanoscale of the samples were also evaluated using a transmission electron microscope (TEM/HRTEM, JEOL 2100F) operating at 200 kV and equipped with a field emission electron gun providing a point resolution of 0.19 nm. The microscope is coupled with an INCA x-sight energy dispersive X-ray spectrometer (EDXS, Oxford Instruments) used for chemical analysis. Additional chemical analyses were performed by inductively coupled plasma-optical emission spectroscopy (ICP-OES, PerkinElmer Optima 3300DV).

X-ray absorption near-edge structure spectroscopy (XANES) measurements were carried out at Eu L3 edge at the Spanish CRG beamline BM25A (SpLine) at the European Synchrotron (ESRF), France. X-ray absorption (XAS) spectra were collected in fluorescence mode at 45° incidence and at RT. Signal was measured using a 13 element Si (Li) solid state detector from 2 eV Scientific Instruments. Final spectra represent an average of three X-ray absorption (XAS) scans. The X-ray absorption data were analysed using ATHENA software^35^.

### Luminescent characterization

Optical properties of these materials were investigated by measuring emission and excitation spectra. The photoluminescence spectra of the phosphor particles were recorded with a spectrofluorometer (Fluorolog®-3, HORIBA Jobin Yvon) at RT. The emission spectrum was measured over the wavelength 400–650 nm, a Xenon arc lamp was used as excitation source (λ_exc_ = 380 nm). The decay profiles were also recorded using the same instrument after the samples were exposed to Solar Simulator, indoor artificial light and monochromatic light, λ_exc_ = 380 nm, for about 10 minutes. A relationship has been established between the luminescence intensity obtained by the spectrofluorometer and the luminace (cd/m^2^) obtained by using a luminance meter (LS-110, Konica Minolta Sensing, Inc.) and verifying the relationship following the approach by Clabau *et al*.^36^. The radioluminescence measurements were obtained exciting the powders with Cu X-ray tube source on Rigaku Ultima+RINT2000/PC diffractometer and light collection was done using an Ocean Optics HR2000 spectrometer equipped with an optical fiber, the spectra were recorded at RT.

## Electronic supplementary material

Supporting Info_Manuscript SREP-17-00105-A
