# Supplementary material for: Precise Tuning of the Nanostructured Surface leading to the Luminescence Enhancement in SrAl2O4 Based Core/Shell Structure
Source: Sci Rep. 2017 Mar 28;7:462. doi: 10.1038/s41598-017-00541-w (PMC5428670; doi:10.1038/s41598-017-00541-w)
Supplement: Supplementary file 1 — Supporting Info_Manuscript SREP-17-00105-A [file 41598_2017_541_MOESM1_ESM.pdf]

Supplementary Material

Precise Tuning of the Nanostructured Surface leading to the Luminescence Enhancement in $SrAl_2O_4$ Based Core/Shell Structure

Rocío Estefanía Rojas-Hernandez[a*], Fernando Rubio-Marcos[a], Aida Serrano[b], Adolfo del Campo[a], José Francisco Fernandez [a]

[a] *Electroceramic Department, Instituto de Cerámica y Vidrio, CSIC, Kelsen 5, 28049, Madrid, Spain.*

[b] *SpLine, Spanish CRG Beamline at the ESRF,F-38043 Grenoble, Cedex 09 France and Instituto de Ciencia de Materiales de Madrid, CSIC, Cantoblanco, 28049, Madrid, Spain*

[*] Corresponding author: Tlf: +34 91 735 58 40; Ext 1074. Fax: +34 91 735 58 43. E-mail address: rociorojas@icv.csic.es (R.E. Rojas-Hernandez)

Supplementary Information 1:

The chemical composition of the commercial powder was determined by X-ray Fluorescence (XRF). X-ray fluorescence analyses were carried out using a Spectrometer (Model MagiX PW 2424, Philips, Eindhoven, Netherlands) with rhodium X-ray tube, 2.4 kW generator, and five-position crystal charger. The spectrometer is also equipped with the IQ analytical software for analysis, which includes fundamental parameters for interelemental correction calculations. The operating conditions were LIF200 and PE analyzing crystals, no primary beam filter, 150 µm sample collimator, vacuum atmosphere, and flow-proportional and scintillation detector. The content of all oxides was determined using the melting method with $Li_2B_4O_7$. For that, 0.3 g of dry samples and 5.5 g of $Li_2B_4O_7$ were homogeneously mixed and placed inside a pellet box and were compacted using a hydraulic press for 1 min at a pressure of 5.1 × 103 kg. Pellets were stored inside desiccators to avoid hydration from air humidity.

Analyzed Composition of commercial powder based on *$SrAl_2O_4$:Eu,Dy* (content of all oxides in wt. %) is summarized in Table 1. This table collects composition of the powder, which was estimated by X-Ray Fluorescence (XRF). ″

Table S1. Analyzed Composition of commercial powder based on *$SrAl_2O_4$:Eu,Dy*

Oxides	Composition (wt%)
**SrO**	48.05
**Al_2O_3**	49.6
**Eu_2O_3**	1.47
**Dy_2O_3**	0.88

Supplementary Information 2:

The SrO-Al_2O_3 system is complex due to the larger number of polymorphic transformations that occur during the heat treatment. The optimization of the conditions of the synthesis is still a technological problem because of a lack of a synthesis procedure to provide a monophasic compound. Moreover, the stability domain is not restricted to a specific temperature range and the crystalline phases present during the synthesis change as function of the kind of precursors and the synthesis route. Employing an α-alumina with an average particle size, $d_{50} \approx 6$ µm using Al_2O_3 /SrO molar ratio of 1, it can be seen from XRD graph that the main phase formed is $SrAl_2O_4$ joined with strontium rich phase such $Sr_{12}Al_{14}O_{33}$ as secondary phase. By SEM characterization it could be observed the growth of nanocrystals on the surface of the platelets. The morphology of the synthesized phase retains the shape of the less soluble reactant, the alumina, so the reaction mechanism is related to the template mechanism formation, where the formation strontium aluminate phase initiates on the Al_2O_3 platelet surface. This type of mechanism is determined by the dissolution rate of the reactants in the molten salt, eutectic mixture *$(NaCl\text{-}KCl)_e$* in this case the mobility of Sr^{2+} and Al^{3+} cations. The movement of Al^{3+} cations is insignificant regarding Sr^{2+} in the molten flux. The less diffusion of Sr^{2+} cations implies the formation of a nanostructured $SrAl_2O_4$ coating on the Al_2O_3 platelet surface. This effect has been mainly described in metals to generate core–shell structure by coating a desired material or its precursor on the surface of the template and its well-known as Kirkendall-effect. This $SrAl_2O_4$ coating formation is associated with remains of Al_2O_3 that have not reacted. Therefore, initial $SrCO_3$ has not been consumed during the reaction. This issue can be correlated to the formation of the strontium rich phase, $Sr_{12}Al_{14}O_{33}$. Therefore, in order to obtain mainly $SrAl_2O_4$, the optimization of Al_2O_3 /SrO ratio was evaluated.

Figure S1 shows the XRD difractograms of the synthesized powders using Al_2O_3/SrO molar ratio of 1, 1.25, 1.43, 1.66 and 2. The molar unit of strontium aluminate phase depends on the different sources of alumina and Al_2O_3 /SrO molar ratio. Here it is essential to note that the stoichiometric relation promotes the formation of the strontium rich phase of SrO-Al_2O_3 system. However, the secondary phase, $Sr_{12}Al_{14}O_{33}$, decreases when the content of SrO decreases. Therefore, the reduction of the SrO content in the nominal stoichiometric composition leads to promote $SrAl_2O_4$ phase formation, decreasing the quantity of raw materials, SrO and rare earths, in the synthesis.

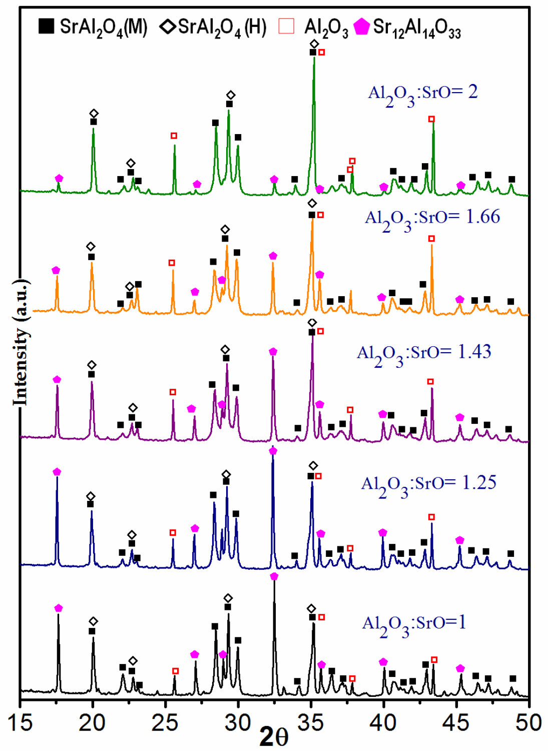

Figure S1. XRD pattern of synthesized $SrAl_2O_4$:Eu, Dy phosphor heated at 1000°C for 2 h in $90N_2$-$10H_2$ atmosphere, employing a salt/$SrAl_2O_4$ molar ratio of 3:1, as precursor alumina platelets with an average particle size, $d_{50} \approx 6$ µm) and using Al_2O_3 /SrO molar ratio of 1, 1.25, 1.43, 1.66 and 2.

Supplementary Information 3:

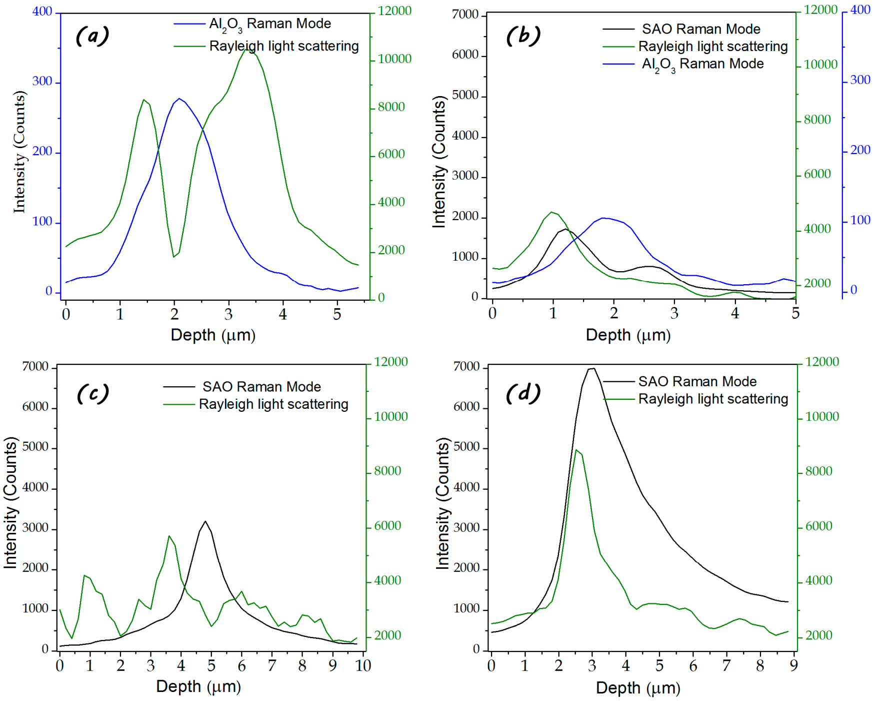

Figure S2. Raman-intensity depth profiles (blue line for 418 cm^{-1} Raman mode of Al_2O_3 and black line for the 467 cm^{-1} Raman mode of $SrAl_2O_4$:Eu, Dy) and Rayleigh light scattering (green line) along the laser incident direction for different samples (a) 6 μm Al2O3 ,particles. (b) Core-shell of $SrAl_2O_4$: Eu, Dy platelets by the molten salt method using Al_2O_3 /SrO molar ratio of 2 . (c) Agglomerates of $SrAl_2O_4$:Eu, Dy nanoparticles obtained from the molten salt method[1] (d) As received Reference microparticle $SrAl_2O_4$:Eu, Dy from Jinan Chenghao Technology Co., Ltd SAO particles.

In order to understand the role of the morphology on the photoluminescence efficiency, a complementary Raman study was carried out. A correlation of Raman intensity signal with optical and emission properties has been observed. This information is valuable for discussion on light-emission mechanism of this material.

To perform this study, four different type of particles were studied: alumina hexagonal platelets (mean particle size: 6 μm); reference micro particle of $SrAl_2O_4$:Eu, Dy material which is formed by particles above 20 μm, core-shell of $SrAl_2O_4$:Eu, Dy platelets (mean particle size: 10 μm) and an agglomerate of $SrAl_2O_4$:Eu, Dy nanoparticles obtained by the molten salt method [1].

Raman study was carried out by means depth Raman spectra to show how varying the Raman intensity of each peak inside the particles.

The particles were dispersed and settled into a glass slide; Figure **S2(a)** shows the depth profile alumina platelets based on the intensity of the 418 cm^{-1} (blue line) and the laser peak (green line). The intensity of the Rayleigh light scattering of the laser increases at the surface of the particle and decreases when the focal plane enters into the middle of the particle. After that a similar behaviour takes place when the laser goes through the half of the particle. Al_2O_3 absorbs the laser energy. Therefore, the intensity of the Rayleigh light scattering of the laser is reduced in the core of the particle. Afterwards, two maximums are observed corresponding to the backside surface of Al_2O_3 platelet because it is supported on the glass slide where the Al_2O_3 platelet has been dispersed. On the contrary the intensity of the 418 cm^{-1} peak increases until achieves the half of the platelet and decreases when crosses the backside of the particle.

The depth profile of Core-shell of SrAl2O4:Eu, Dy platelets is illustrated in **Figure S2(b)** based on the intensity of the 418 cm^{-1} (A_{1g}) Raman Mode, 467 cm^{-1} attributed to the bending of O-Al-O bonds of $SrAl_2O_4$ phase and the Rayleigh light scattering of the laser. Once again, the intensity of the Rayleigh light scattering of the laser

increases at the surface ≈ 0.2 μm before particle surface and decreases going through the particle due to the attenuation by $SrAl_2O_4$. Concerning the intensity of 467 cm^{-1} peak, the depth profile shows two maximum corresponding to the outer layer of $SrAl_2O_4$:Eu, Dy. The intensity of the 418 cm^{-1} achieves the maximum in the inner part of the platelet in which Al_2O_3 is confined. The depth profiles corresponding to the peaks located at 467 cm^{-1} and 418 cm^{-1} corroborate effectively the shell-like formation of $SrAl_2O_4$ and the core-like presence of Al_2O_3.

Regarding the agglomerates of $SrAl_2O_4$:Eu, Dy nanoparticles (**Fig. S2(c))**, the depth profiles related to the peak located at 467 cm^{-1} of $SrAl_2O_4$ phase and the Rayleigh light scattering of the laser have a symmetric shape, the up and down slope is almost equal.

Furthermore, bulk $SrAl_2O_4$:Eu, Dy particles were analysed; It is important to remark that these particles are formed completely by $SrAl_2O_4$, the 418 cm^{-1} (A_{1g}) Raman Mode was not observed. **Figure S2(d)** illustrates the depth profile of the 467 cm^{-1} (black line) and the Rayleigh light scattering of the laser (green line). The intensity of the Rayleigh light scattering of the laser increases until ≈ 0.2 μm before particle surface and decreases due to the mitigation by $SrAl_2O_4$. The variation of the intensity of 467 cm^{-1} peak has similar behavior with the 418 cm^{-1} peak; achieving the maximum in the particle surface and decreasing slowly going through the particle, because of the size of the particles, the intensity attenuation goes over > 9 μm. In spite of the larger dimension the laser light could not reach the center of the particle (particle diameter is ca.20 μm and after 6 μm the green light did not reach the surface, so there is a part of the particle that does not contributed to the optical response in the visible region).

Rayleigh light scattering of the laser is measured in term of net counts by using the same laser powder. The differences in counts indicates that the more homogenous surface of reference $SrAl_2O_4$:Eu, Dy microparticles reflect more efficiently the coherent light than the nanostructured ones. Both the core-shell and the agglomerate of nanoparticles have a maximum of light reflection that is less than 50% of the correspondent to the reference particles. The roughness of nanostructured material produces a higher diffuse scattering of the light.

Raman intensities are measured in terms of counts; the varying in intensity of 467 cm^{-1} Raman Mode can be related to the material volume. For this reason the counts acquired in the depth profile of SrAl2O4:Eu, Dy platelets are lower than in bulk $SrAl_2O_4$:Eu, Dy particles and agglomerates; being most intense in bulk sample. These findings suggest that the outer $SrAl_2O_4$:Eu, Dy layer in core-shell structure is ≤ 500 nm.

Supplementary Information 4:

There is not a standardized way of measuring the life time, called phosphorescence decay time or afterglow time. Different regulations have been employed to evaluate the photoluminescence of commercial products employing as source Xe Arc Lamp (1000 lux). However, the majority of decay curves are taken when the sample is irradiated with monochromatic light at 350, 365, 375 nm and 380 for 5 and 10 min [2–9] and by a solar simulator in a lesser extent[10]. For this reason, the powders obtained have been irradiated by different sources. Also, the decay curves have been obtained for all the powders synthesized at 1000ºC compared with the commercial powder as received.

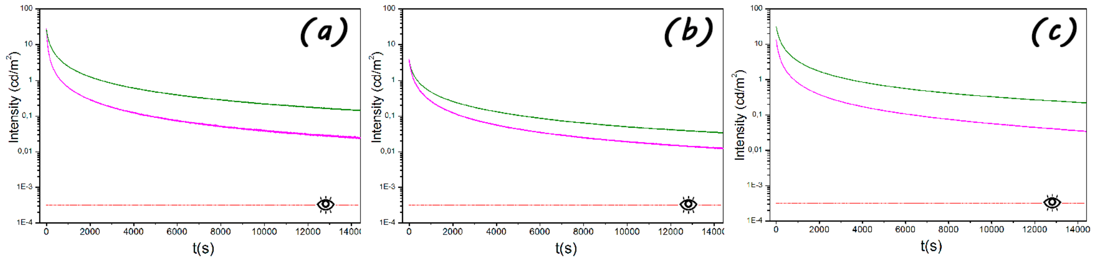

Figure S3 shows the afterglow decay curve of $SrAl_2O_4$:Eu, Dy powder synthesized at 1000ºC during 2 hour in $90N_2$-$10H_2$ atmosphere, employing a salt/$SrAl_2O_4$ molar ratio of 3:1, as precursor alumina platelets with an average particle size, $d_{50} \approx 6$ µm, (magenta decay curve) and using Al_2O_3/SrO molar ratio of 2 compared to the commercial powder (green decay curve) after the light source was cut off, irradiated for 10 min by **(a)** monochromatic light at 380 nm, **(b)** artificial indoor illumination and **(c)** solar simulator.

Supplementary Information 5:

When irradiated with X-rays, the $SrAl_2O_4$: Eu,Dy powder scintillates emitting visible light.[11] To get some evaluation of the radioluminescence response of $SrAl_2O_4$: Eu,Dy powder, radioluminescence spectra for the powder synthesized at 1000ºC for 2 h in $90N_2$-$10H_2$ atmosphere, employing a salt/$SrAl_2O_4$ molar ratio of 3:1 employing as precursor alumina platelets with an average particle size, $d_{50} \approx 6$ μm) and using Al_2O_3 /SrO molar ratio of 2 (magenta spectrum) are shown in **Figure S4.**

The intense of broad peak at 525 nm is associated with Eu^{2+} 5d4f→4f transitions and indicates the presence of reduced europium in the sample. A less intense peak at 616 nm is associated to Eu^{3+} $^5D_0 \rightarrow {}^7F_2$ transition.[12] These results indicate that there are divalent and trivalent charged states inside the matrix. With the further increasing alumina average particle size, its intensity smoothly increases, being the $SrAl_2O_4$: Eu, Dy platelets which show the better response. The same behavior has been observed when the powders are excited by monochromatic light at 380 nm.

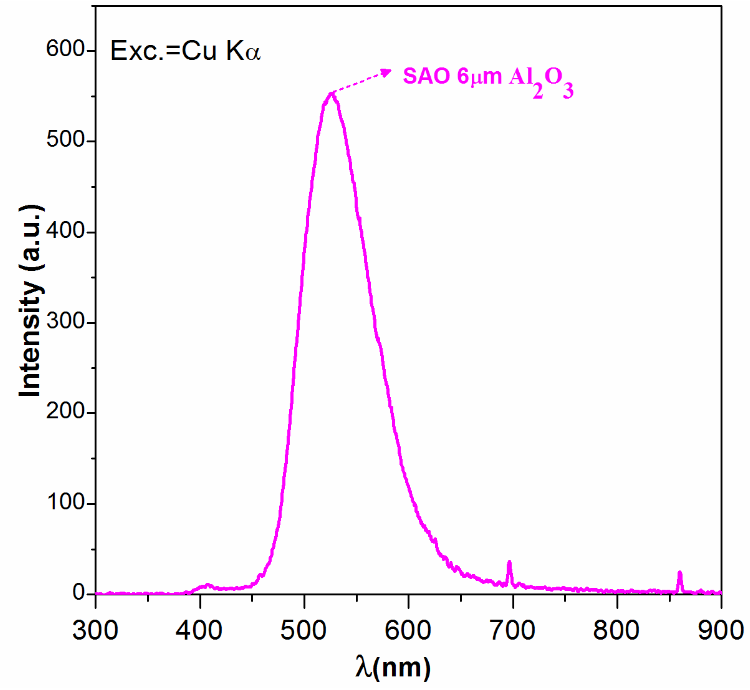

Figure S4. Radioluminescence spectra of the powder synthesized at 1000ºC for 2 hour in $90N_2$-$10H_2$ atmosphere, employing a salt/$SrAl_2O_4$ molar ratio of 3:1, as precursor alumina platelets with an average particle size, $d_{50} \approx 6$ μm) and using Al_2O_3 /SrO molar ratio of 2.

Supplementary Information 6:

X-ray absorption spectroscopy (XAS) measurements at the Eu L_3 edge were performed in order to quantify the relative amounts of the two oxidation states of Europium. Reference spectra of Eu^{2+} (EuI) and Eu^{3+} (Eu_2O_3) are shown in **Figure S5a** (bottom panel). A significant difference in the energy can serve as a signature of the two oxidation states (6971.78 for Eu $^{2+}$ and 6979.47 for Eu $^{3+}$). The spectra of the powder synthesized at 1000 (magenta spectrum), 1200 (blue spectrum), 1400 ºC (purple spectrum) for 2 hour in $90N_2$-$10H_2$ atmosphere, employing a salt/$SrAl_2O_4$ molar ratio of 3:1, as alumina platelets with an average particle size, $d_{50} \approx 6$ µm) and Al_2O_3 /SrO molar ratio of 2 and initial reference material: microparticle $SrAl_2O_4$:Eu, Dy from Jinan Chenghao Technology Co., Ltd (green spectrum) are shown in Figure **S5a** (top panel). These spectra consist of two peaks coinciding with the reference spectra of Eu^{2+} and Eu^{3+}, definitively confirming the coexistence of both oxidation states in samples. High-temperature annealing results in a notable reduction in the Eu^{3+} and an increase in Eu^{2+} peak intensity.
The relative amounts of Eu^{2+} for different temperature annealing in synthesized $SrAl_2O_4$: Eu, Dy powders were determined from the deconvoluted peak fitting using a pseudo-Voigt and arctangent step functions [13,14]. The fitted curve (red dotted curve) and the individual fitted components (green, blue and orange dotted curves) are shown in the same graph (**Fig. S5b**) and the fitting results of the characteristic white lines of Eu^{2+} and Eu^{3+} are listed in **Table S2.**

Quantification of relatively abundance of the different ions in Eu compounds usually is usually done without taking into account transition probabilities of the two states[15]. However, we take the greatest reported transition probability difference between L_3-edge absorption intensity for adjusting the Eu^{2+} and Eu^{3+} peak intensity. A factor R=1.5 has employed becasuse in our sample the Eu content is 0.14 mol% (the system is considered dilute as the Eu content is less than 3 mol %) [13,15]. Moreover, it is known that the area ratio A_{Eu} between the Eu^{2+} and Eu^{3+} WLs is related to the mole ratio M_{Eu} through $M_{Eu}=RA_{Eu}$.[13,16]. Therefore in our study, we use an R value of 1.5 to determine M_{Eu}, which is used to calculate the mole fraction of Eu ions, the result is listed in the final column of Table S2.

A fitting of the experimental XANES spectra was also performed by means of a linear combination of the reference ones (Eu_2O_3 and EuI) (Table S3). The relative amounts of Eu^{2+} determined by two fitting methods, for different $SrAl_2O_4$: Eu,Dy powders processed here, are shown in Figure S5c. Eu fraction results are qualitatively similar, showing the same trend: Eu^{2+} fraction increases as synthesis annealing temperature increases. Although the trend is similar in both XANES fitting, we obtained best R-factor (goodness of fit parameter) values for the results given from deconvoluted peak fitting. Thus, those results are presented together % of the photoluminescence in order to analyze the linked relationship with Eu^{2+} fraction in Figure 3(d) in the main document.

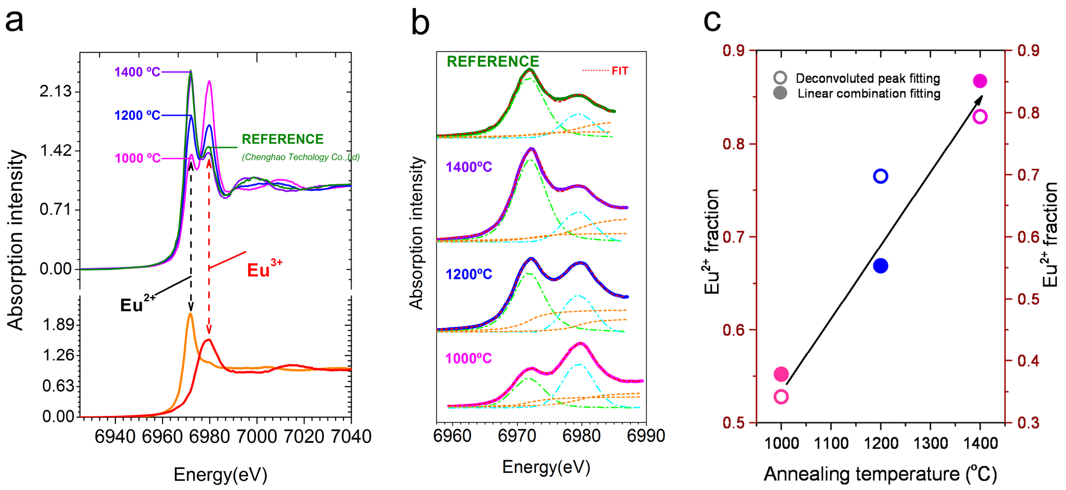

Figure S5. (a) Top panel: XANES spectra of the powder synthesized at 1000 (magenta spectrum), 1200 (blue spectrum), 1400 ºC (purple spectrum) during 2 hour in $90N_2$-$10H_2$ atmosphere, employing a salt/$SrAl_2O_4$ molar ratio of 3:1 employing as alumina platelets with an average particle size, $d_{50} \approx 6$ µm) and using Al_2O_3 /SrO molar ratio of 2 and initial reference material: microparticle $SrAl_2O_4$:Eu, Dy from Jinan Chenghao Technology Co., Ltd (green spectrum). Bottom panel: XANES reference spectra of Eu^{2+} (EuCl) and Eu^{3+} (Eu_2O_3). **(b)** In order to quantify the white-line intensity (WL) in these spectra, curve fitting is accomplished by modeling the experimental data using pseudo-Voigt for the white-line resonances and arctan for the edge steps functions. **(c)** Eu^{2+} fraction obtained from two fitting routes (deconvoluted peak and linear combination linear) for sample synthesized at 1000, 1200, and 1400 ºC.

Table S2. Fitting results of Eu^{2+} and Eu $^{3+}$ L3 WLs from the powder synthesized at 1000, 1200 ,1400 ºC during 2 hour in $90N_2$-$10H_2$ atmosphere, employing a salt/$SrAl_2O_4$ molar ratio of 3:1 employing as alumina platelets with an average particle size, $d_{50} \approx 6$ µm and using Al_2O_3 /SrO molar ratio of 2 and initial reference material: microparticle $SrAl_2O_4$:Eu, Dy from Jinan Chenghao Technology Co., Ltd by deconvoluted peak fitting.

		Edge Resonance				Edge step			
Sample	Species	Height a.u.	Position eV	FWHM	Area	Height a.u.	Position eV	Fraction	R-factor
1000ºC	Eu^{2+}	7.45	6971.78	5.87	7.45	0.418	6971.78	0.528	0.00019
	Eu^{3+}	9.97	6979.47	5.71	9.97	0.589	6979.47	0.472	
1200ºC	Eu^{2+}	11.66	6971.78	5.87	11.66	0.581	6971.78	0.765	0.00015
	Eu^{3+}	5.38	6979.47	5.68	5.38	0.352	6979.47	0.235	
1400ºC	Eu^{2+}	15.57	6971.78	5.84	15.57	0.228	6971.78	0.829	0.00060
	Eu^{3+}	4.81	6979.47	5.79	4.81	0.673	6979.47	0.171	
Reference material	Eu^{2+}	15.57	6971.78	5.85	15.57	0.581	6971.78	0.8160	0.00012
	Eu^{3+}	5.38	6979.47	5.78	5.38	0.352	6979.47	0.1840	

Table S3. Fitting results of Eu^{2+} and Eu $^{3+}$ L3 WLs from the powder synthesized at 1000, 1200, 1400 ºC during 2 hour in $90N_2$-$10H_2$ atmosphere, employing a salt/$SrAl_2O_4$ molar ratio of 3:1 employing as alumina platelets with an average particle size, $d_{50} \approx 6$ µm and using Al_2O_3 /SrO molar ratio of 2 and initial reference material: microparticle $SrAl_2O_4$:Eu, Dy from Jinan Chenghao Technology Co., Ltd by linear combination fitting.

Sample	Species	Fraction	R-factor
1000ºC	Eu^{2+}	0.378	0.0938
	Eu^{3+}	0.622	
1200ºC	Eu^{2+}	0.553	0.0674
	Eu^{3+}	0.447	
1400ºC	Eu^{2+}	0.851	0.0405
	Eu^{3+}	0.149	
Reference material	Eu^{2+}	0.831	0.0589
	Eu^{3+}	0.169	

Supplementary Information 7:

Figure S6 (a). shows the XRD patterns of synthesized $SrAl_2O_4$:Eu, Dy phosphor heated at 1000, 1200, and 1400 °C for 2 h in $90N_2$-$10H_2$ atmosphere, employing a salt/$SrAl_2O_4$ molar ratio of 3:1 employing as precursor alumina platelets with an average particle size, $d_{50} \approx 6$ µm) and using Al_2O_3 /SrO molar ratio of 2. **Figure S6 (b).** shows the Raman Shift image of the Raman mode attributed to the bending of O-Al-O bonds of $SrAl_2O_4$ phase. The Raman imaging allows us evaluating the homogeneity on the sample because each pixel on the image comprises a full Raman spectrum. The statistical analysis of the O-Al-O mode Raman shift of all spectra in the image is shown in Fig. **S5**(b). The maximum Raman shift (468 cm^{-1}, marked in red) and the minimum Raman shift (465cm^{-1}, marked in purple).

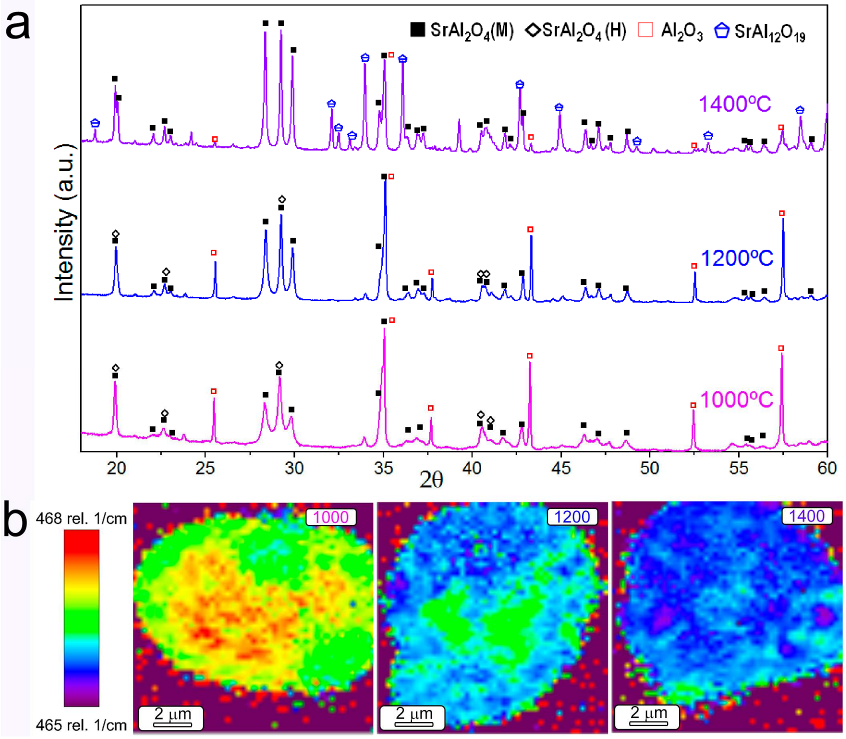

Figure S6. (a) XRD patterns of synthesized $SrAl_2O_4$:Eu, Dy phosphor heated at 1000, 1200, and 1400 °C for 2 h in $90N_2$-$10H_2$ atmosphere, employing a salt/$SrAl_2O_4$ molar ratio of 3:1 employing as precursor alumina platelets with an average particle size, $d_{50} \approx 6$ µm) and using Al_2O_3 /SrO molar ratio of 2. (b) Raman Shift image of the Raman mode attributed to the bending of O-Al-O bonds of $SrAl_2O_4$ phase of synthesized $SrAl_2O_4$:Eu, Dy phosphor heated at 1000, 1200, and 1400 °C.

Supplementary Information 8:

The morphological nature of the as received commercial powder used can be seen from **Figure S7a-c**. The powder is formed by particles ranging from 8 to 40 μm; the particles show an irregular shape. In **Figure S7b**, it can be observed that the particles are dense and have sharp fracture surfaces that resembles mirrow surface as consequence of previous grinding process. A more exhaustive inspection of the morphology reveals that at the surface small grains having hundreds of nanometers are presents. The surface nanostructured seems to be form a smooth layer by a reaction after the grinding; see **Fig. S7c.** The low magnification TEM image (**Fig. S7(d))** shows the presence of large and dense particles with a mean particle size of ca. 16 μm.

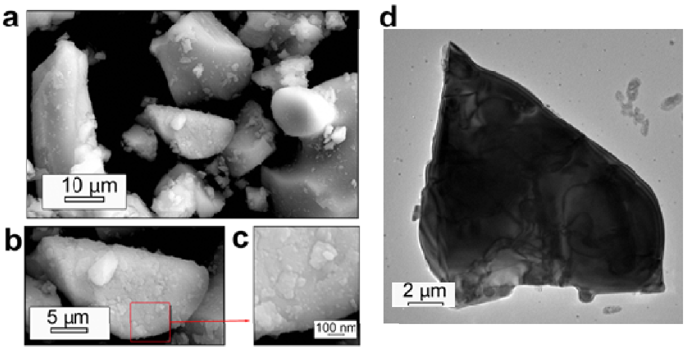

Figure S7. The morphological nature of the as received commercial powder: (a) FE-SEM micrographs of commercial $SrAl_2O_4$ powders with sizes of 8-40 μm; **(b)** Larger dense particle; **(c)** Detail of the particle that reveals a highly compacted structure having a nanostructured surface; (d) low magnification TEM image.

Supplementary References

1. Rojas-Hernandez, R. E. *et al.* Original Synthetic Route To Obtain a $SrAl_2O_4$ Phosphor by the Molten Salt Method: Insights into the Reaction Mechanism and Enhancement of the Persistent Luminescence. *Inorg. Chem.* **54,** 9896–9907 (2015).

2. Chen, I.-C. *et al.* Investigations into thermoluminescence and afterglow characterization of strontium aluminates with boron-modification and reductions via sol-gel route. *J. Rare Earths* **30,** 972–978 (2012).

3. Son, N. M., Vien, L. T. T., Ngoc, L. V. K. B., Trac, N. N. & Trac. Synthesis of $SrAl_2O_4$:Eu^{2+} , Dy $^{3+}$ phosphorescence nanosized powder by combustion method and its optical properties. *Mater. Sci.* **187,** (2009).

4. Xiao, Q., Xiao, L., Liu, Y., Chen, X. & Li, Y. Synthesis and luminescence properties of needle-like $SrAl_2O_4$:Eu, Dy phosphor via a hydrothermal co-precipitation method. *J. Phys. Chem. Solids* **71,** 1026–1030 (2010).

5. Nag, A. & Kutty, T. R. N. Role of B_2O_3 on the phase stability and long phosphorescence of $SrAl_2O_4$:Eu, Dy. *J. Alloys Compd.* **354,** 221–231 (2003).

6. Haranath, D., Shanker, V., Chander, H. & Sharma, P. Studies on the decay characteristics of strontium aluminate phosphor on thermal treatment. *Mater. Chem. Phys.* **78,** 6–10 (2002).

7. Zhu, Y., Zheng, M., Zeng, J., Xiao, Y. & Liu, Y. Luminescence enhancing encapsulation for strontium aluminate phosphors with phosphate. *Mater. Chem. Phys.* **113,** 721–726 (2009).

8. Kshatri, D. S. & Khare, A. Comparative study of optical and structural properties of micro- and nanocrystalline $SrAl_2O_4$:Eu^{2+}, Dy^{3+} phosphors. *J. Lumin.* **155,** 257–268 (2014).

9. Dejene, F. B., Kebede, M. a., Redi-Abshiro, M. & Kgarebe, B. V. Structural and photoluminescence properties of Dy^{3+} co-doped and Eu^{2+} activated MAl_2O_4 (M=Ba,Ca,Sr) nanophosphors. *Opt. Mater. (Amst).* **35,** 1927–1931 (2013).

10. Kaya, Y., Karacaoglu, E. & Bekir, Lk. Effect of Al/Sr ratio on the luminescence properties of $SrAl_2O_4$:Eu^{2+}, Dy^{3+} phosphors. *Ceram. Int.* **38,** 3701–3706 (2012).

11. Montes, P. J. R., Valerio, M. E. G. & Rezende, M. V. do. S. Mechanisms of radioluminescence of rare earths doped $SrAl_2O_4$ and $Ca_{12}Al_{14}O_{33}$ excited by X-ray. *J. Electron Spectros. Relat. Phenomena* **189,** 39–44 (2013).

12. Ayvacikli, M., Ege, A. & Can, N. Radioluminescence of SrAl2O4 :Ln^{3+} (Ln = Eu, Sm, Dy) phosphor ceramic. *Opt. Mater. (Amst).* **34,** 138–142 (2011).

13. Henke, B., Palick, C., Keil, P., Johnson, J. A. & Schweizer, S. Eu oxidation state in

fluorozirconate-based glass ceramics. *J. Appl. Phys.* **106,** (2009).

14. Brugger, J. *et al.* The oxidation state of europium in hydrothermal scheelite: In situ measurement by XANES spectroscopy. *Can. Mineral.* **44,** 1079–1087 (2006).

15. Rakovan, J., Newville, M. & Sutton, S. Evidence of heterovalent europium in zoned llallgua apatite using wavelength dispersive XANES. *Am. Mineral.* **86,** 697–700 (2001).

16. Antonio, M. R., Soderholm, L. & Song, I. Design of spectroelectrochemical cell for in situ X-ray absorption fine structure measurements of bulk solution species. *J. Appl. Electrochem.* **27,** 784–792 (1997).
